# Supplementary figures and images for: Neuroticism alters the transcriptome of the frontal cortex to contribute to the cognitive decline and onset of Alzheimer’s disease
Source: Transl Psychiatry. 2021 Feb 24;11:139. doi: 10.1038/s41398-021-01253-6 (PMC7904919; doi:10.1038/s41398-021-01253-6)

## A Conscientiousness

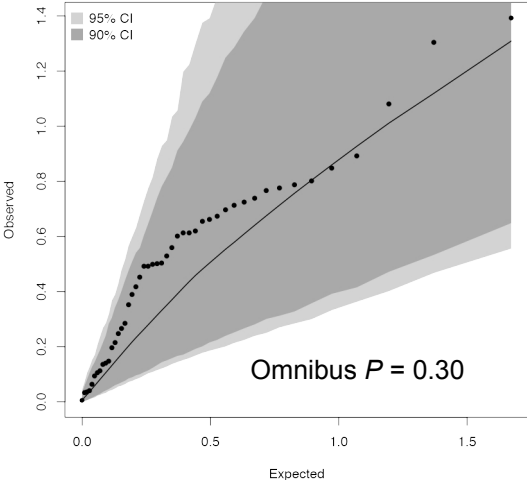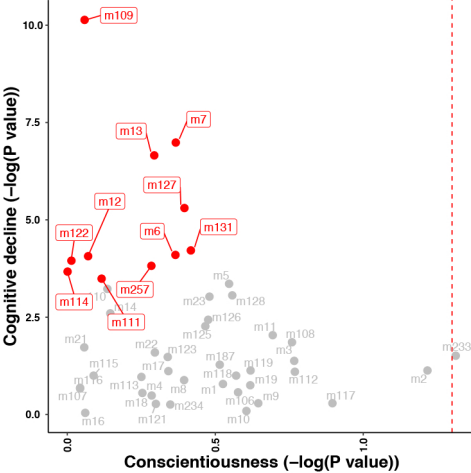

## B Agreeableness

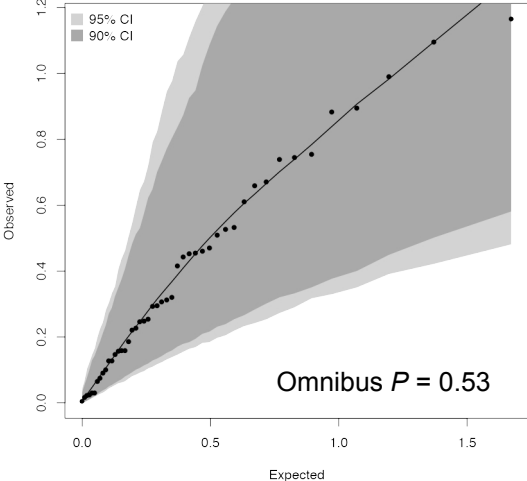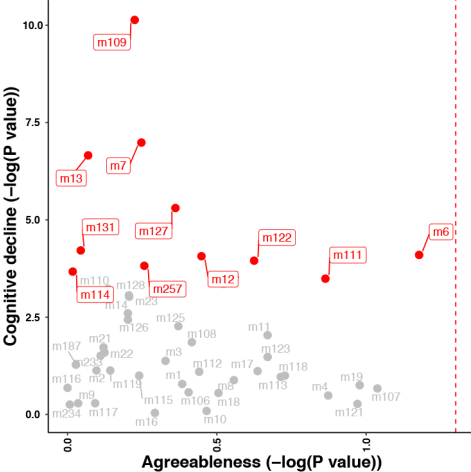

### C Openness

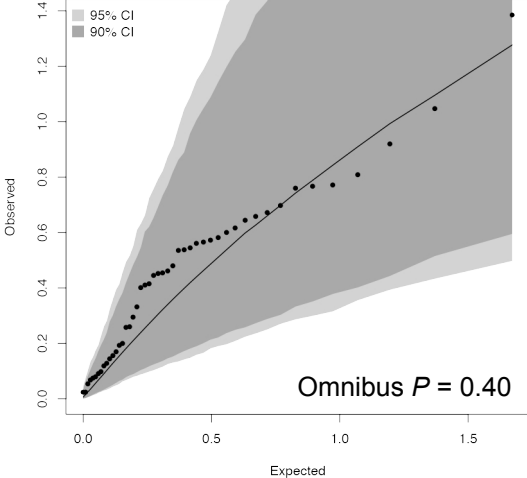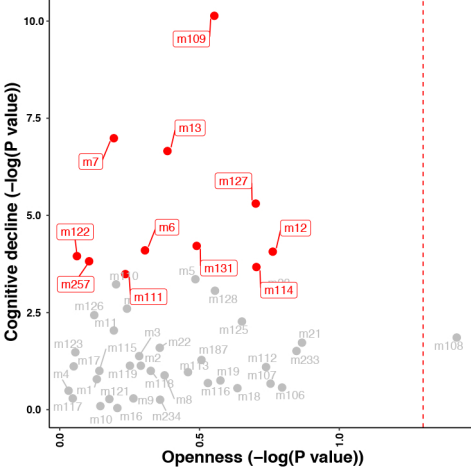

## D Extraversion

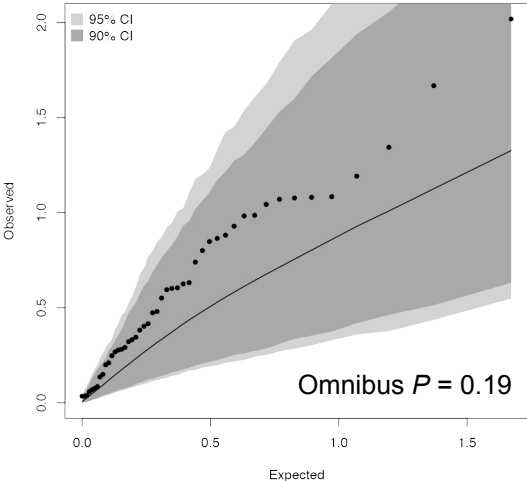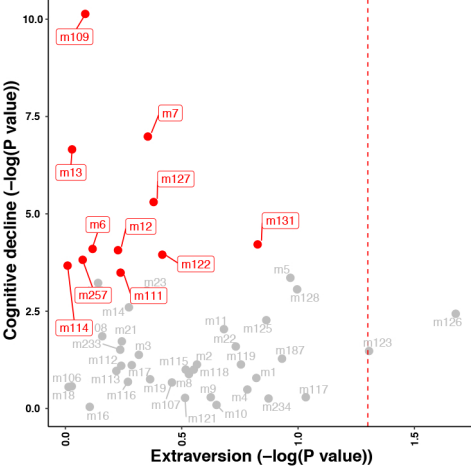

Supplement: Supplementary file 2 — Supplementary Figure 1 [file 41398_2021_1253_MOESM2_ESM.pdf]

A

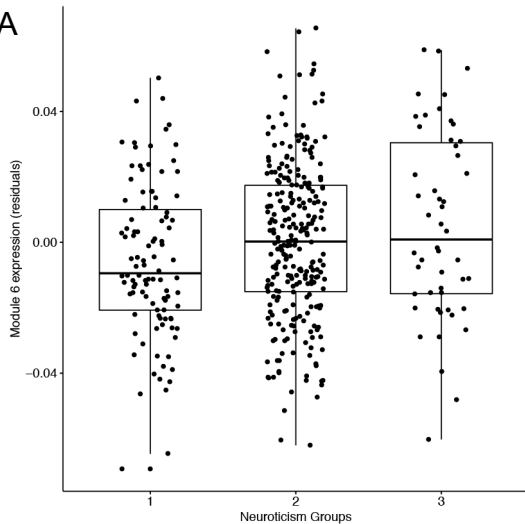

B

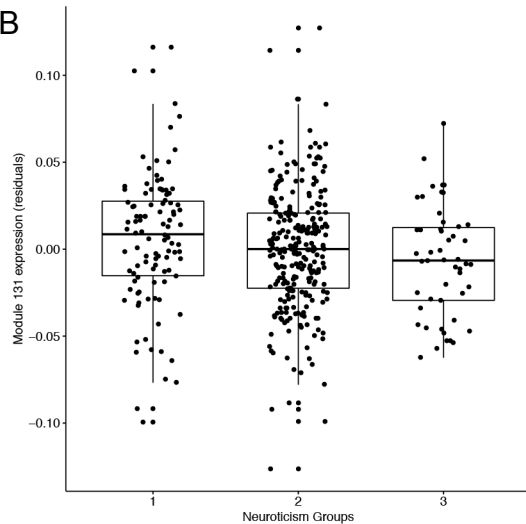

Supplement: Supplementary file 3 — Supplementary Figure 2 [file 41398_2021_1253_MOESM3_ESM.pdf]
